# Supplementary material for: The interactome of intact mitochondria by cross-linking mass spectrometry provides evidence for coexisting respiratory supercomplexes
Source: Mol Cell Proteomics. 2017 Dec 8;17(2):216–32. doi: 10.1074/mcp.RA117.000470 (PMC5795388; doi:10.1074/mcp.RA117.000470)
Supplement: Supplemental Data [file supp_RA117.000470_133922_0_supp_23978_fzffwf.zip › spectra_annotation/mito_spectra_annotation/382-1-6-1-6-1.pdf]

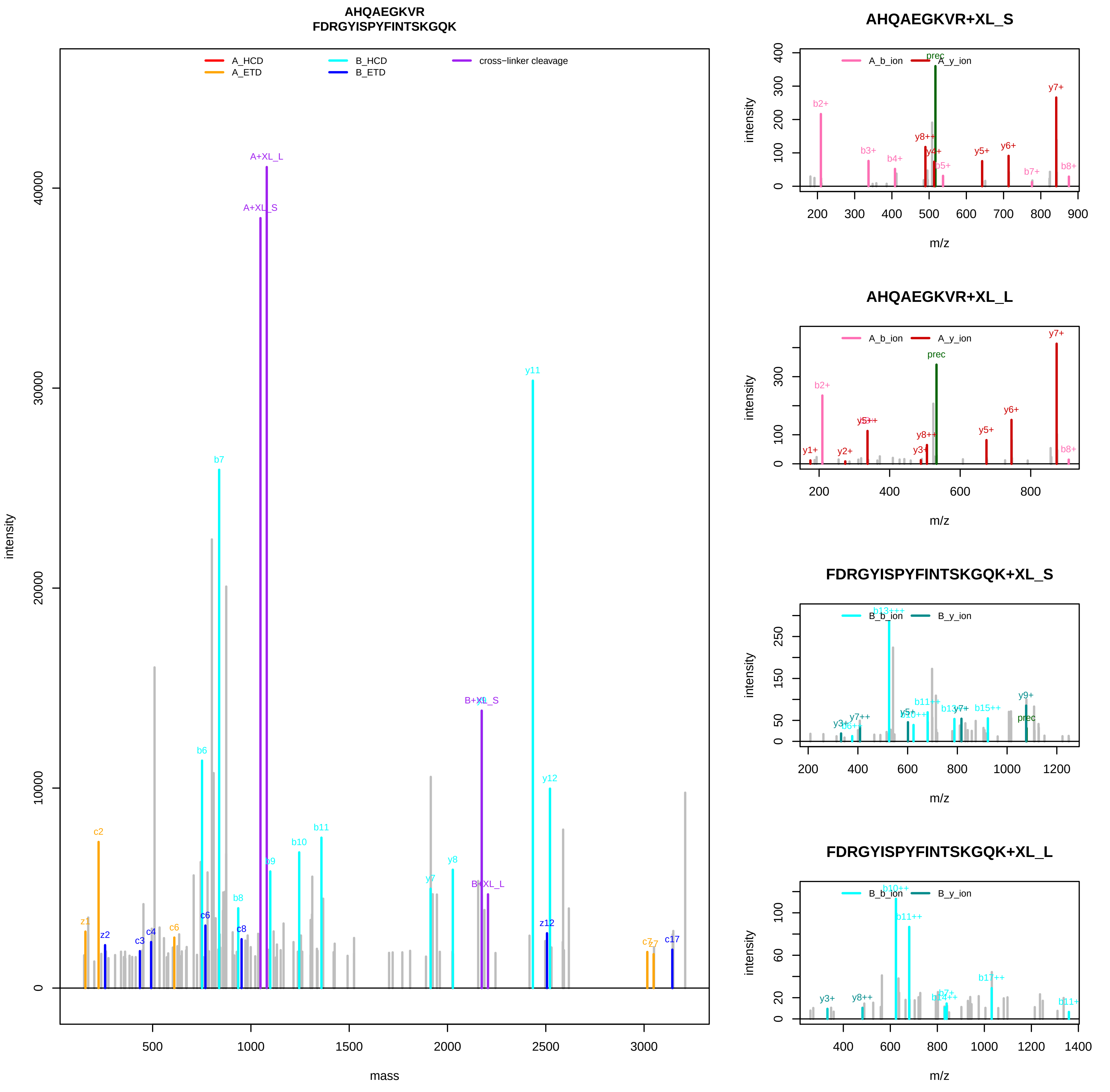

AHQAEGKVR
FDRGYISPYFINTSKGQK
A_HCD
A_ETD
B_HCD
B_ETD
cross−linker cleavage
A+XL_L
A+XL_S
B+XL_S
B+XL_L
y11
b7
b6
y12
b11
b10
b9
y8
y7
b8
c2
z1
z2
c3
c4
c6
c8
z12
c7
z7
c17
intensity
mass
0
10000
20000
30000
40000
500
1000
1500
2000
2500
3000
AHQAEGKVR+XL_S
A_b_ion
A_y_ion
prec
b2+
b3+
b4+
b5+
b7+
b8+
y4+
y5+
y6+
y7+
y8++
m/z
AHQAEGKVR+XL_L
y1+
y2+
y3+
y5+
y6+
y7+
y8++
b2+
b8+
FDRGYISPYFINTSKGQK+XL_S
B_b_ion
B_y_ion
b13+++
b11++
b13++
b15++
b10++
y3+
y5+
y7+
y7++
y9+
FDRGYISPYFINTSKGQK+XL_L
b10++
b11++
b17++
b14++
b7+
b11+
y3+
y8++
